# Dissection of Biological Property of Chinese Acupuncture Point Zusanli Based on Long-Term Treatment via Modulating Multiple Metabolic Pathways

**DOI:** 10.1155/2013/429703

**Published:** 2013-09-01

**Authors:** Guangli Yan, Aihua Zhang, Hui Sun, Weiping Cheng, Xiangcai Meng, Li Liu, Yingzhi Zhang, Ning Xie, Xijun Wang

**Affiliations:** National TCM Key Lab of Serum Pharmacochemistry, Key Lab of Chinmedomics and Key Pharmacometabolomics Platform of Chinese Medicines, First Affiliated Hospital, Heilongjiang University of Chinese Medicine, Heping Road 24, Harbin 150040, China

## Abstract

Acupuncture has a history of over 3000 years and is a traditional Chinese medical therapy that uses hair-thin metal needles to puncture the skin at specific points on the body to promote wellbeing, while its molecular mechanism and ideal biological pathways are still not clear. High-throughput metabolomics is the global assessment of endogenous metabolites within a biologic system and can potentially provide a more accurate snap shot of the actual physiological state. We hypothesize that acupuncture-treated human would produce unique characterization of metabolic phenotypes. In this study, UPLC/ESI-HDMS coupled with pattern recognition methods and system analysis were carried out to investigate the mechanism and metabolite biomarkers for acupuncture treatment at “Zusanli” acupoint (ST-36) as a case study. The top 5 canonical pathways including *alpha*-linolenic acid metabolism, d-glutamine and d-glutamate metabolism, citrate cycle, alanine, aspartate, and glutamate metabolism, and vitamin B6 metabolism pathways were acutely perturbed, and 53 differential metabolites were identified by chemical profiling and may be useful to clarify the physiological basis and mechanism of ST-36. More importantly, network construction has led to the integration of metabolites associated with the multiple perturbation pathways. Urine metabolic profiling might be a promising method to investigate the molecular mechanism of acupuncture.

## 1. Introduction 

**Figure 1:**
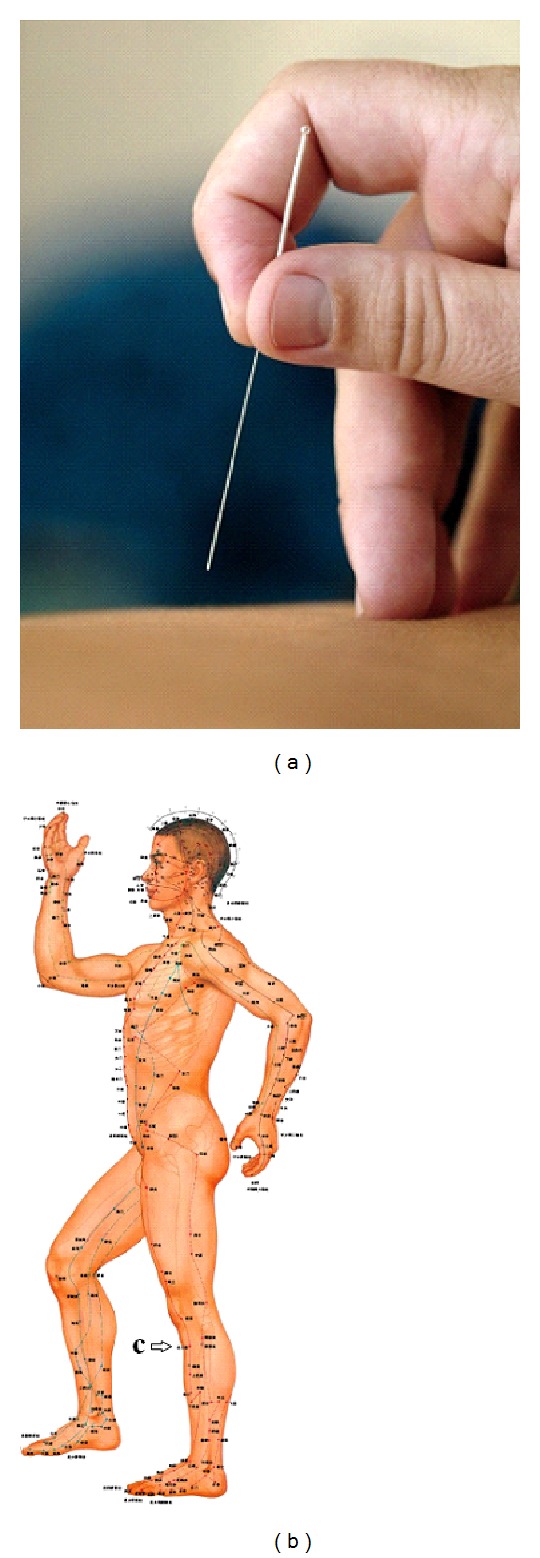
Acupuncture (a) and acupoint map of human meridians (b). c: “Zusanli” acupoint (ST-36).

Acupuncture (Figure 1(a)), which utilizes fine needles to pierce through specific anatomical points (called “acupoints”), is an ancient traditional Chinese medical therapy that is used widely around the world [1]. The National Institutes of Health has defined acupuncture as “a family of procedures involving stimulation of anatomical locations on the skin by a variety of techniques” [2]. President Richard Nixon's visit to China in 1972 was the seminal event opening the door to Chinese medical practices. Since that time, there has been a growing interest in integrating acupuncture into Western medical practice. On November 16, 2010, acupuncture has been listed by UNESCO as “Intangible Cultural Heritage.” Acupuncture uses a unique holistic approach to cure human diseases through establishment of equilibrium in the human life, body, mind, intellect, and soul, so satisfactory treatment results could be achieved [3–5]. Therapeutic effects of acupoint stimulation primarily work through 14 principal meridians (Figure 1(b)) that represent channels through which energy known as Qi flows [6]. Nowadays, this traditional Chinese medical technique has become very popular worldwide as a complementary medicine, and some of the common applications of acupuncture include heart disease, smoking cessation, and the treatment of inflammatory diseases and psychological disorders [7–10].

Acupuncture is a safe, traditional Chinese medical therapy that is often perceived as both calming and relaxing for patients. Human studies have found a physiological basis for acupuncture needling that involves both central and peripheral networks [11]. This understanding of the anatomical and physiologic nature of acupuncture points and meridians remains insufficient, and the challenge is therefore to clarify the true meaning. Although basic research on acupuncture has made considerable progress in the past 10 years, we still lack a clear picture of “how acupuncture works.” We have to understand the traditional acupuncture and present it in the light of the 21st century scientific thoughts and experimental evidence. Metabolomics can capture global changes and overall physiological status in biochemical networks and pathways in order to elucidate sites of perturbations and has shown great promise as a means to identify biomarkers of disease [12–15]. It has become practically available and resembles acupuncture in many aspects that serves as the major driving force for translation of acupuncture medicine revolution into practice [16]. Metabolomics and acupuncturology are of some similar characteristics such as entirety, comprehensiveness, and dynamic changes. The area of integrating acupuncture with metabolomics approach has become a major hot of TCM research and will advance paving the road towards health care [17].

In traditional Chinese medicine (TCM), Zusanli (also known as ST-36, Figure 1(b)c) point of “The Stomach Meridian of Foot-Yangming” is commonly used in human acupuncture to treat a wide range of health conditions including gastrointestinal disorders such as stomach ache, abdominal pain and distension, constipation, diarrhea, vomiting, dysentery, indigestion, and others [18, 19]; however, little is understood about its biological basis and mechanism. Herein, the present study aims to gain a better insight into acupuncture-treated ST-36; low molecular-weight metabolite data were analyzed to detect the enriched clusters, to determine the perturbation pathways and to infer the biological processes. The specific and unique biochemical pathways can be identified and greatly facilitated by using multivariate statistical analysis methods.

## 2. Experimental Procedures

### 2.1. Chemicals and Reagents

Acetonitrile (HPLC grade) was purchased from Merck (Germany), respectively; the distilled water was produced by a Milli-Q ultrapure water system (Millipore, Billerica, USA); formic acid was of HPLC grade and obtained from Kermel Chemical Reagent Co., Ltd., China; leucine enkephalin was purchased from Sigma-Aldrich (St. Louis, MO, USA). All other reagents were of analytical grade.

### 2.2. Ethical Statement and Subjects

Written informed consents were obtained from all subjects. The experimental protocol was reviewed and approved by the Ethical Committee of Heilongjiang University of Chinese Medicine (no. HLJZY-2012-0198) and was conducted according to the principles expressed in the Declaration of Helsinki. Control subjects (*n* = 20) with a mean age ± SD of 25.4 ± 4.2 years were enrolled in this study from Heilongjiang University of Chinese Medicine, China. Acupuncture stimulation was performed on bilateral ST-36 for 30 min, once a day for two weeks. The outcomes of Health Survey Questionnaire were assessed, and the related clinical information including gender, age, body mass index, and main parameters was collected in Supplementary Table 1 (see Supplementary Material available online at http://dx.doi.org/10.1155/2013/429703).

### 2.3. Sample Preparation

The urine samples were collected at day points, and the endogenous metabolites were analyzed by an ultraperformance liquid chromatography-quadrupole time-of-flighthigh-definition mass spectrometry (UPLC-Q-TOF-HDMS). The subjects were given insulated ice packs in which they were asked to store the urine samples immediately until they were received by the study investigator. On arrival at the laboratory, the samples were centrifuged at 10,000 rpm for 10 min at 4°C to remove any solid debris. Fractions (10 mL) of the urine supernatants were then stored at −80°C until UPLC-Q-TOF-HDMS analysis. Thawed urine samples were collected after centrifugation at 13,000 rpm for 15 minutes at 4°C, and the supernatant was transferred to a 1.5 mL polypropylene tube and then filtered through a syringe filter (0.22 *μ*m); 5 *μ*L of the supernatant was injected into the UPLC-Q-TOF-HDMS.

### 2.4. Metabolic Profiling and Metabolite Analysis

Chromatography was carried out with an ACQUITY UPLC BEH C_18_ column (100 mm × 2.1 mm, 1.7 *μ*m) using an ACQUITY UPLC system (Waters Corp., Milford, USA). A “purge-wash-purge” cycle was employed on the autosampler, with 90% aqueous formic acid used for the wash solvent and 0.1% aqueous formic acid used as the purge solvent; this ensured that the carryover between injections was minimized. The column was maintained at 50°C, and subsequently a gradient of 0.1% formic acid in acetonitrile (solvent A) and 0.1% formic acid in water (solvent B) was used as follows: a linear gradient of 0-1 min, 2% A; 1–5 min, 2–10% A; 5–7 min, 10–22% A; 7–11 min, 22–24% A; 11–16 min, 24–35% A; 16–18 min, 35–100% A. The flow rate was 0.50 mL/min, and 2 *μ*L aliquot of each sample was injected into the column. The eluent was introduced to the mass spectrometry directly, that is, without a split. Quality control samples were used to minimize the analytical variation, evaluate the compound stability, and monitor the sample preparation process. After every 10 sample injections, a pooled sample followed by a blank was injected in order to ensure consistent performance of the system.

The eluent was introduced into the Synapt High-Definition MS (Waters Corp., Milford, USA) analysis, and the optimal conditions were as follows: desolvation temperature of 350°C, source temperature of 110°C, sample cone voltage of 30 V, extraction cone voltage of 3.5 V for positive ion mode and 4.0 V negative ion mode, collision energy of 4 eV, microchannel plate voltage of 2400 V, cone gas flow of 50 L/h and desolvation gas flow of 700 L/h, and capillary voltage of 3.2 kV for positive ion mode and 2.6 kV negative ion mode. Centroid data was acquired between m/z 50 and 1000 using an accumulation time of 0.2 s per spectrum. For accurate mass acquisition, a lock mass of leucine enkephalin at a concentration of 200 pg/mL was used via a lock spray interface at a flow rate of 100 *μ*L·min^−1^ monitoring for positive ion mode ([M + H]^+^ = 556.2771) and negative ion mode ([M − H]^−^ = 554.2615) to ensure accuracy during the MS analysis.

The MassFragment application manager was used to facilitate the MS/MS fragment ion analysis process by way of chemically intelligent peak-matching algorithms. The identities of the specific metabolites were confirmed by comparison of their mass spectra and chromatographic retention times with those obtained using commercially available reference standards. This information was then submitted for database searching, either in-house or using the online ChemSpider database and MassBank data source.

### 2.5. Multivariate Data Analysis

Centroid and integrated raw mass spectrometric data were processed using MassLynx V4.1 and MarkerLynx software (Waters Corp., Milford, USA). The intensity of each ion was normalized with respect to the total ion count to generate a data matrix that consisted of the retention time, m/z value, and the normalized peak area. The multivariate data matrix was analyzed by EZinfo software (Waters Corp., Milford, USA). The unsupervised segregation was checked by principal components analysis (PCA) using pareto-scaled data. With PCA, data were visualized by plotting the PC scores where each point in the scores plot represents an individual sample and the PC loadings where each point represents one mass/retention time pair. Potential markers of interest were extracted from S plots constructed following analysis with orthogonal partial least squares to latent structures discriminant analysis (OPLS-DA), and markers were chosen based on their contribution to the variation and correlation within the data set.

### 2.6. Construction and Analysis of Metabolic Pathway

The construction, interaction, and pathway analysis of potential biomarkers were performed with MetPA based on database source including the KEGG (http://www.genome.jp/kegg/) and Human Metabolome Database (http://www.hmdb.ca/) to identify the affected metabolic pathways analysis and visualization. Subsequently, the possible biological role was evaluated by the enrichment analysis using the MetaboAnalyst. 

### 2.7. Statistical Analyses

SPSS 17.0 for Windows was used for the statistical analysis. The data were analysed using the Wilcoxon Mann-Whitney *U* Test, with *P* < 0.05 set as the level of statistical significance.

## 3. Results 

### 3.1. Metabolomic Profiling

For UPLC-MS analysis, aliquots were separated using a Waters Acquity UPLC (Waters, Milford, MA) and analyzed using a Q-TOF/HDMS, which consisted of an electrospray ionization source and linear ion-trap mass analyzer. The UPLC-MS representative profiles of consecutively injected samples of the same aliquot showed stable retention time with no drift in all of the peaks (Supplementary Figure 1). The stable profiles reflected the stability of UPLC-HDMS analysis and reliability of the metabolomic data. Low molecular mass metabolites could be separated well in the short time of 18 min due to the minor particles (sub-1.7 *μ*m) of UPLC.

### 3.2. Pattern Recognition Analysis

**Figure 2:**
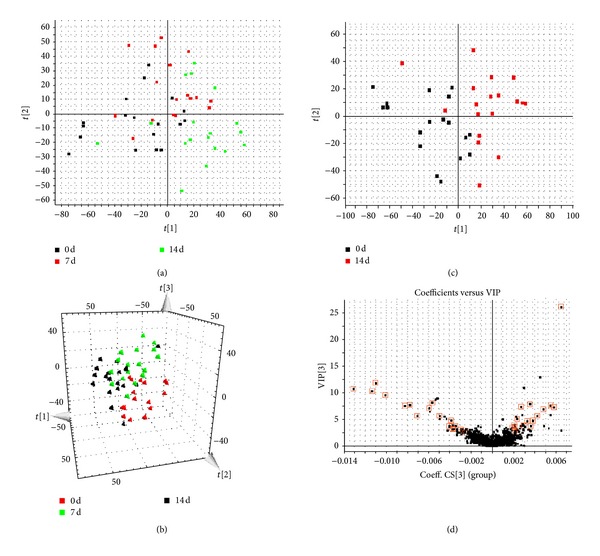
PCA model results for control and model group in positive mode (a). Trajectory analysis of PCA score plots (3D) for the serum samples in positive mode (b). PCA model results for 0 day and 14 days in positive mode (c). VIP plot of OPLS-DA of samples in positive mode (d).

**Figure 3:**
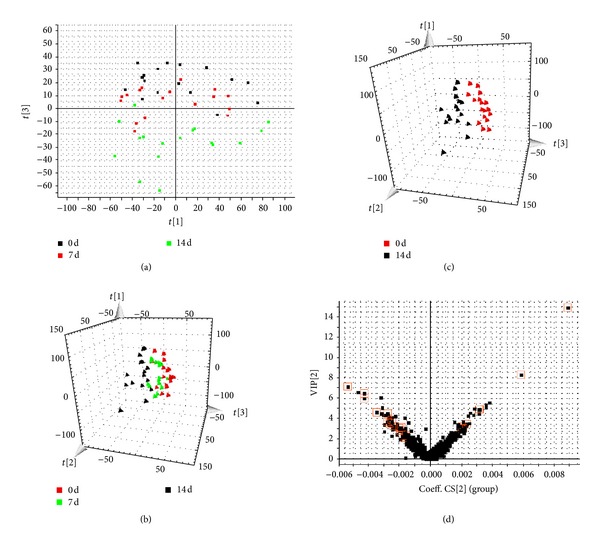
PCA model results for control and model group in negative mode (a). Trajectory analysis of PCA score plots (3D) for the serum samples in negative mode (b). PCA model results for 0 day and 14 days in negative mode (c). VIPplot of OPLS-DA of samples in negative mode (d).

Both multivariate projection approaches such as PCA and OPLS-DA often can be taken, because of their ability to cope with highly multivariate, noisy, collinear, and possibly incomplete data. The PCA score plots showed that the metabolic profiles of the 7 and 14 days significantly changed as a result of acupuncture treatment (Figures 2(a) and 3(a)). Trajectory analyses of the urine samples in the three-dimensional score plots were corresponding to Figure 1(b) in positive mode and Figure 2(b) in negative mode. The ions that showed significant difference in abundance between the 0-day and 14-day treated humans were contributed to the observed separation (Figures 2(c) and 3(c)) and selected from the respective S plots as potential markers in positive and negative modes (Figures 2(d) and 3(d)). Overall 8236 retention time-exact mass pairs were determined in metabolomic profile of urine samples. VIP-value threshold cutoff of the metabolites was set to 2.0 and, above this threshold, were filtered out as potential biomarkers. Finally, the markers of significant contribution were characterized 30 in positive mode and 23 in negative mode (Table 1). 53 metabolites were identified and verified by reference standards among the differential metabolites. From the above plots, various metabolites could be identified as being responsible for the separation between 0-day and 14-day groups and were therefore viewed as potential biomarkers.

### 3.3. Identification of Important Differential Metabolites

**Figure 4:**
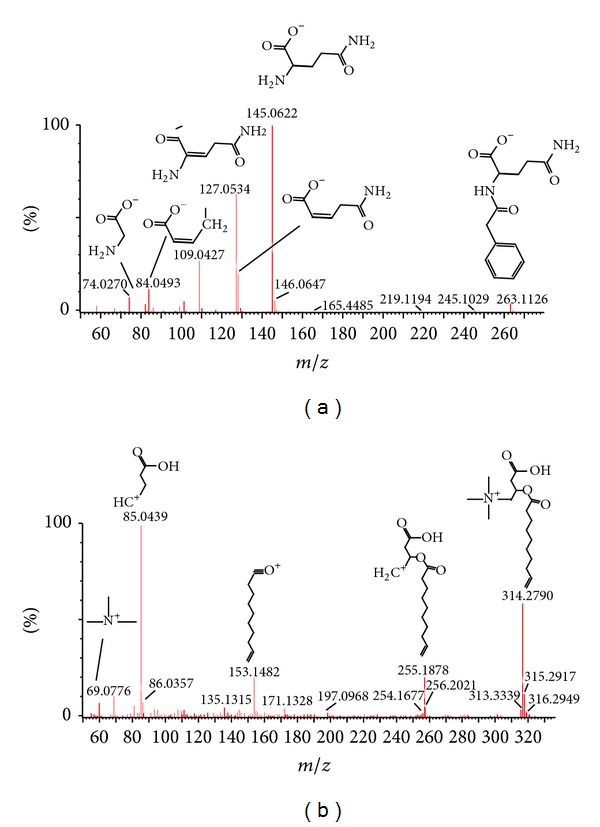
Mass fragment information of *alpha*-N-phenylacetyl-L-glutamine in negative mode (a) and 9-decenoylcarnitine in positive mode (b).

The robust UPLC-HDMS analysis platform provides the retention time, precise molecular mass, and MS/MS data for the structural identification of biomarkers. The molecular mass was determined within measurement errors by Q-TOF, and meanwhile the potential elemental composition, degree of unsaturation, and fractional isotope abundance of compounds were also obtained. The presumed molecular formula was searched in ChemSpider, Human Metabolome Database, and other databases to identify the possible chemical constitutions, and MS/MS data were screened to determine the potential structures of the ions. According to the protocol detailed above, 53 endogenous metabolites contributing to the separation of the model group and control group were detected in the samples (Table 1). Monitoring changes of these metabolites may predict the development of acupuncture-treated. Therefore, these metabolites were selected as candidate markers for further validation. Taking an example, the precise molecular weight is 263.1126, and the main fragment ions that were analyzed via the MS/MS screening and were observed in the negative ion spectrum. Its calculated molecular formula was speculated to be C_13_H_16_N_2_O_4_ based on the analysis of its elemental composition and fractional isotope abundance; the ion was inferred to be *alpha*-N-phenylacetyl-L-glutamine. Its mass spectrum is illustrated in Figure 4(a). The precise molecular weight is 314.2790, and the main fragment ions that were analyzed via the MS/MS screening were observed in the positive ion spectrum (Figure 4(b)). Its calculated molecular formula was speculated to be C_17_H_31_NO_4_, and the ion was inferred to be 9-decenoylcarnitine.

### 3.4. Metabolic Pathway and Function Analysis

**Figure 5:**
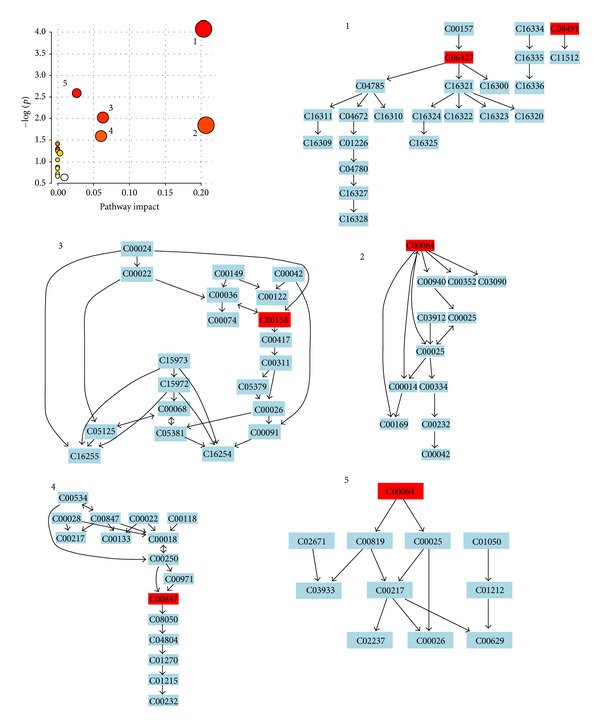
Construction of the arachidonic acid metabolism pathways in acupuncture-treated human. The map was generated using the reference map by KEGG (http://www.genome.jp/kegg/). The green boxes: enzymatic activities with putative cases of analogy in acupuncture-treated human. 1: *alpha*-linolenic acid metabolism; 2: D-glutamine and D-glutamate metabolism; 3: citrate cycle (TCA cycle); 4: alanine, aspartate, and glutamate metabolism; 5: vitamin B6 metabolism.

More detailed analyses of pathways and networks influenced by acupuncture were performed by MetPA which is a free, web-based tool that combines result from powerful pathway enrichment analysis with the topology analysis. Metabolic pathway analysis with MetPA revealed that metabolites which were identified together were important for the host response to acupuncture-treated and were responsible for *alpha*-linolenic acid metabolism, d-glutamine and d-glutamate metabolism, citrate cycle (TCA cycle), alanine, aspartate, and glutamate metabolism, and vitamin B6 metabolism (Figure 5 and Supplementary Table 2). Potential biomarkers were also identified from these relevant pathways. Some significantly changed metabolites have been found and used to explain the arachidonic acid metabolism. The detailed construction of the arachidonic acid metabolism pathways with higher score was shown in Figure 5. These results suggested that these pathways showed the marked perturbations over the entire time course of acupuncture-treated.

## 4. Discussion

Acupuncture has been practiced in China for thousands of years as an important therapeutic method in TCM and has been gradually accepted in Western countries as an alternative or complementary treatment [20]. Acupuncture, a therapeutic modality with few or no adverse effects, is a nonpharmacological therapy in which needles are inserted at specific cutaneous locations of the body, known as acupoints, and has been widely used to reduce some symptoms or to treat diseases in clinical practice [21]. However, little is understood about its biological basis and how the acupuncture works. Extensive studies have been conducted on the mechanism of acupuncture to explain the effects of acupuncture on various systems and symptoms [22, 23]. Biomarkers are indicators of biological processes and pathological states that can reveal a variety of health and disease traits and could facilitate and improve the development of diseases treatments and benefit the public health [24]. It is important to note that the progress in metabolomics technology has provided sensitive, fast and robust tools to analyze biomarkers, within a biologic system [25]. It resembles TCM in many aspects such as study method and design and opens up a novel opportunity to reinvestigate TCM [26]. Therefore, combining metabolomics with in-depth investigations of acupuncture will enable a revolution in our understanding of disease and will advance personalized medicine. However, metabolomics analyses evaluating the physiological basis of acupuncture-treated human have yet not been examined. This study was therefore designed to further elucidate the underlying mechanism of acupuncture-treated man from the metabolic pathways in a global view.

In this study, metabolomic analysis of key regulatory metabolites in acupuncture-treated man was successfully investigated by UPLC-HDMS combined with multivariate statistical analysis. Interestingly, we have identified 53 specific metabolites relevant for ST-36. To examine the effect of the 0-day acupuncture on ST-36, global metabolomic profiles were compared between 0 day and 14 days. In total, 53 metabolites (33 decreased and 20 increased) were significantly changed. Five unique metabolic pathways were also indicated to be differentially affected in acupuncture-treated human. Of note, we found that acupuncture activated an array of factors involved in *alpha*-linolenic acid metabolism, TCA cycle, d-glutamine and d-glutamate metabolism, alanine, aspartate, and glutamate metabolism, and vitamin B6 metabolism pathways. The significantly downregulated and upregulated biomarkers were observed following the 14-day treatment. Metabolic analyses of acupuncture were inferred from changes in the intermediates during substance metabolism. Based on the findings of this study, it would appear that many different metabolic pathways were disrupted as a result of acupuncture on ST-36. In the present study, we describe the physiological basis and biological property of ST-36, which will help us understand the basic mechanisms of acupuncture. Integrated network analysis of the metabolites differentially expressed in acupuncture-treated yields highly related signaling networks and suggests strongly that the involvement of these signaling networks could be essential for the development of acupuncture. In its grandest vision, the application of metabolomics approaches to the study of acupuncture-perturbed networks will, through the identification of therapeutic drug targets, foster a future of personalized medicine. Biologically, several metabolite biomarkers for acupuncture treatment are revealed and serve as the candidates for further mechanism investigation. This topic is in progress as our further direction. Further integration of the data from other levels, such as gene expression and proteomics levels, will improve the robustness of the identified biomarker.

Metabolomics is a rapidly developing field that has given new hope in the treatment of acupuncture. With this change, it appears that all the ancient Chinese concepts can be reinterpreted and harmonized with the latest finding in metabolomic research. Application of metabolomic technologies to the study of the acupuncture will increase our understanding of the pathophysiological processes, and this should help us identify potential biomarkers to develop new therapeutic strategies. The rapid growth of metabolomics field provides an array of new tools for the integration of acupuncture with modern technology and systems biology and is potentially advancing the progress of modernization and internationalization of acupuncture [27–29]. More work needs to be performed to explore the molecular mechanisms underlying acupuncture, and this could lead to novel treatments to maximize the therapeutic benefits of acupuncture. Acupuncture medicine can take advantage of metabolomic framework to provide the healthcare system with useful tools that can optimize the effectiveness of treatment. Researchers believe that metabolomics has the potential to revolutionize the practice of acupuncture medicine, improve the diagnosis, prevention, and treatment of disease, and reduce adverse drug reactions.

## 5. Conclusions

In order to realize the potential role of acupuncture, an intrinsic part of TCM, metabolomics technologies are being increasingly used for acupuncture medicine; efforts are in progress in major therapeutic areas. Furthermore, because of the dynamic nature of the metabolome and its responsiveness to perturbations associated with stimuli, greater understanding will be needed in key metabolites or metabolic pathways toward the elucidation of molecular mechanisms underlying. We have firstly constructed the metabolomic feature profiling and metabolite interaction network of ST-36 by using validated pattern recognition approach and ingenuity pathways analysis. Analyzing the topology of the network, we have detected 53 potential biomarkers and predicted the major metabolites network of ST-36. Combining the results from these methods, we have calculated five high confidence networks. The identified target metabolites were found to encompass a variety of pathways. The power of metabolomics to capture and elucidate metabolic characters of the ST-36 has been successfully demonstrated in this study. Our study also highlights the importance of metabolomics as a potential tool for uncovering metabolic pathways to assess acupuncture treatment. We expect that, in future, metabolomics would serve as “Trojan horses in the 21st century” in the field of acupuncture treatment.

## Supplementary Material

Supplementary Figure S1: UPLC-MS BPI urine chromatograms of acupuncture-treated human in positive mode (A) and negative mode (B).
Supplementary Table 1: Clinical characteristics of the healthy subjects at baseline.
Supplementary Table 2: Result from ingenuity pathway analysis.

## Figures and Tables

**Table 1:** Potential biomarkers identified of acupuncture-treated human in positive and negative modes.

No.	Rt (min)	*m*/*z* determined	*m*/*z* calculated	Error (mDa)	Ion form	Formula	Metabolite name	VIP value	Trend	*P* value
1	0.50	162.1126	162.1130	−0.4	[M + H]^+^	C_7_H_15_NO_3_	Carnitine	5.1	↑	0.0087
2	2.32	290.1597	290.1604	−0.7	[M + H]^+^	C_13_H_23_NO_6_	3-Methylglutarylcarnitine	4.8	↑	0.0431
3	3.45	276.1815	276.1811	0.4	[M + H]^+^	C_13_H_25_NO_5_	Hydroxyhexanoycarnitine	3.4	↑	0.0327
4	4.77	265.1205	265.1188	1.7	[M + H]^+^	C_13_H_16_N_2_O_4_	*alpha*-N-Phenylacetyl-L-glutamine	9.0	↓	0.0010
5	4.86	316.1761	316.1760	0.1	[M + H]^+^	C_15_H_25_NO_6_	Derivative of carnitine	3.8	↑	0.0426
6	5.48	300.1818	300.1811	0.7	[M + H]^+^	C_15_H_25_NO_5_	Derivative of carnitine	4.6	↑	0.0000
7	5.55	318.1922	318.1917	0.5	[M + H]^+^	C_15_H_27_NO_6_	Derivative of carnitine	7.2	↑	0.0366
8	5.71	135.0433	135.0446	−1.1	[M + H]^+^	C_8_H_6_O_2_	6Z-Octene-2,4-diynoic acid	8.1	↓	0.0230
9	5.98	225.1089	225.1103	−1.4	[M + Na]^+^	C_10_H_18_O_4_	Sebacic acid	2.0	↓	0.0000
10	6.29	302.1969	302.1981	−1.2	[M + H]^+^	C_15_H_27_NO_5_	Derivative of carnitine	5.7	↑	0.0039
11	7.17	284.1844	284.1862	−1.8	[M + H]^+^	C_15_H_25_NO_4_	Supinine	6.9	↑	0.0039
12	7.65	161.0599	161.0603	−0.4	[M + H]^+^	C_10_H_8_O_2_	1-Formyl-2-indanone	3.0	↓	0.0263
13	7.68	354.2273	354.2280	−0.7	[M + H]^+^	C_19_H_31_NO_5_	Derivative of carnitine	3.5	↑	0.0015
14	7.71	233.1166	233.1154	1.2	[M + Na]^+^	C_12_H_18_O_3_	Jasmonic acid	2.9	↑	0.0083
15	8.00	137.0607	137.0603	0.4	[M + H]^+^	C_8_H_8_O_2_	3-Vinylcatechol	6.6	↑	0.0019
16	8.08	330.2264	330.2280	−4.8	[M + H]^+^	C_17_H_31_NO_5_	6-Keto-decanoylcarnitine	11.8	↓	0.0048
17	8.17	286.2018	286.2018	0.0	[M + H]^+^	C_15_H_27_NO_4_	2-Octenoylcarnitine	26.1	↑	0.0000
18	8.31	332.2440	332.2437	0.3	[M + H]^+^	C_17_H_33_NO_5_	Derivative of carnitine	3.9	↑	0.0000
19	8.32	330.2272	330.2280	−0.8	[M + H]^+^	C_17_H_31_NO_5_	6-Keto-decanoylcarnitine	5.7	↓	0.0077
20	10.11	356.2424	356.2437	−1.3	[M + H]^+^	C_19_H_33_NO_5_	Derivative of carnitine	2.3	↓	0.0024
21	10.50	288.2167	288.2175	−0.8	[M + H]^+^	C_15_H_29_NO_4_	L-Octanoylcarnitine	3.9	↑	0.0027
22	10.72	358.2587	358.2593	−0.6	[M + H]^+^	C_19_H_35_NO_5_	Derivative of carnitine	3.7	↓	0.0000
23	12.54	312.2162	312.2175	−1.3	[M + H]^+^	C_17_H_29_NO_4_	2-trans,4-cis-Decadienoylcarnitine	9.5	↓	0.0000
24	12.62	314.2345	314.2331	1.4	[M + H]^+^	C_17_H_31_NO_4_	9-Decenoylcarnitine	3.1	↓	0.0242
25	12.79	312.2169	312.2175	−0.6	[M + H]^+^	C_17_H_29_NO_4_	2-trans,4-cis-Decadienoylcarnitine	10.7	↓	0.0010
26	13.98	338.2312	338.2307	0.5	[M + Na]^+^	C_17_H_33_NO_4_	L-Decanoylcarnitine	7.7	↓	0.0001
27	14.25	338.2310	338.2307	0.3	[M + Na]^+^	C_17_H_33_NO_4_	L-Hexanoylcarnitine	7.6	↑	0.0000
28	14.49	338.2321	338.2307	1.4	[M + Na]^+^	C_17_H_33_NO_4_	L-Decanoylcarnitine	7.5	↓	0.0023
29	15.93	489.2449	489.2464	−1.5	[M + Na]^+^	C_25_H_38_O_8_	Androsterone glucuronide	2.5	↓	0.0171
30	17.02	279.2342	279.2324	1.8	[M + H]^+^	C_18_H_30_O_2_	*alpha*-Linolenic acid	2.5	↓	0.0019
31	0.65	191.0183	191.0192	−0.9	[M − H]^−^	C_6_H_8_O_7_	Citric acid	14.9	↑	0.0005
32	0.76	129.0177	129.0188	−1.1	[M − H]^−^	C_5_H_6_O_4_	Mesaconic acid	8.3	↑	0.0000
33	0.86	199.0080	199.0099	−1.9	[M − H]^−^	C_5_H_12_O_4_S_2_	2-Hydroxypropyl-CoM	3.5	↓	0.0000
34	0.99	232.0275	232.0280	−0.5	[M − H]^−^	C_8_H_11_NO_5_S	Dopamine 3-O-sulfate	2.3	↓	0.0005
35	1.03	161.0444	161.0450	−0.6	[M − H]^−^	C_6_H_10_O_5_	3-Hydroxy-3-methyl-glutaric acid	3.1	↑	0.0000
36	1.11	182.0461	182.0453	0.8	[M − H]^−^	C_8_H_9_NO_4_	4-Pyridoxic acid	2.2	↑	0.0000
37	1.43	253.0827	253.0824	0.3	[M − H]^−^	C_11_H_14_N_2_O_5_	Nicotinamide riboside	3.2	↓	0.0029
38	2.42	225.0860	225.0875	−1.5	[M − H]^−^	C_10_H_14_N_2_O_4_	Porphobilinogen	3.1	↓	0.0000
39	3.49	212.0002	212.0018	−1.6	[M − H]^−^	C_8_H_7_NO_4_S	Indoxyl sulfuric acid	6.0	↓	0.0000
40	4.68	263.0994	263.1032	−3.8	[M − H]^−^	C_13_H_16_N_2_O_4_	*alpha*-N-Phenylacetyl-L-glutamine	3.7	↓	0.0007
41	5.70	187.0048	187.0065	−1.7	[M − H]^−^	C_7_H_8_O_4_S	4-Sulfobenzyl alcohol	4.7	↓	0.0144
42	5.79	193.0353	193.0348	0.5	[M − H]^−^	C_6_H_10_O_7_	Unknown	3.2	↑	0.0001
43	6.21	377.1456	377.1461	−0.5	[M − H]^−^	C_17_H_22_N_4_O_6_	Reduced riboflavin	6.5	↓	0.0001
44	6.39	201.1112	201.1127	−1.5	[M − H]^−^	C_10_H_18_O_4_	Sebacic acid	4.5	↓	0.0000
45	6.55	173.0799	173.0814	−1.5	[M − H]^−^	C_8_H_14_O_4_	Suberic acid	2.8	↓	0.0002
46	7.53	229.1425	229.1440	−1.5	[M − H]^−^	C_12_H_22_O_4_	Dodecanedioic acid	3.9	↓	0.0000
47	7.68	153.0905	153.0916	−1.1	[M − H]^−^	C_9_H_14_O_2_	5,7-Nonadienoic acid	3.0	↑	0.0000
48	7.77	225.1595	225.1603	−0.8	[M − H]^−^	C_12_H_22_N_2_O_2_	1,8-Diazacyclotetradecane-2,9-dione	2.1	↓	0.0000
49	7.78	145.0593	145.0613	−2.0	[M − H]^−^	C_5_H_10_N_2_O_3_	L-Glutamine	7.2	↓	0.0015
50	7.96	185.1181	185.1178	0.3	[M − H]^−^	C_10_H_18_O_3_	10-oxo-decanoic acid	3.2	↓	0.0000
51	8.06	141.0901	141.0916	−1.5	[M − H]^−^	C_8_H_14_O_2_	Unknown	4.8	↑	0.0000
52	8.20	199.0956	199.0970	−1.4	[M − H]^−^	C_10_H_16_O_4_	Decenedioic acid	4.9	↑	0.0016
53	9.09	179.1069	179.1072	−0.3	[M − H]^−^	C_11_H_16_O_2_	3-tert-Butyl-5-methylcatechol	3.5	↑	0.0009
