# Supplementary material for: Dissection of Biological Property of Chinese Acupuncture Point Zusanli Based on Long-Term Treatment via Modulating Multiple Metabolic Pathways
Source: Evid Based Complement Alternat Med. 2013 Sep 1;2013:429703. doi: 10.1155/2013/429703 (PMC3773888; doi:10.1155/2013/429703)
Supplement: Supplementary file 1 — Supplementary Figure S1: UPLC-MS BPI urine chromatograms of acupuncture-treated human in positive mode (A) and negative mode (B). Supplementary Table 1: Clinical characteristics of the healthy subjects at baseline. Supplementary Table 2: Result from ingenuity pathway analysis. [file 429703.f1.doc]

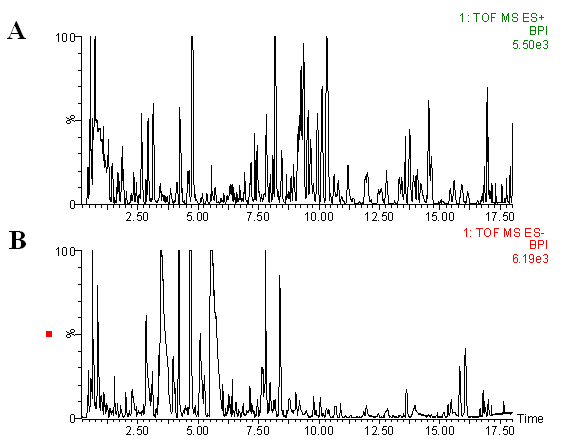


**S Figure-1** UPLC-MS BPI urine chromatograms of acupuncture-treated human in positive mode (A) and negative mode (B).

**Supplementary Table 1**. Clinical characteristics of the healthy subjects at baseline

| **Samples** | **Control** |
| --- | --- |
| Sample No. | 20 |
| Age | 25.4 ± 4.2 |
| BMI(kg/m2) | 22.31±3.72 |
| ALT (U/L) | 71.92±32.65 |
| AST (U/L) | 56.30±39.73 |
| Total bilirubin (mg/dL) | 2.83 ± 1.66 |
| Direct bilirubin(mg/dL) | 1.47±1.08 |
| Indirect bilirubin(mg/dL) | 0.81 ± 0.59 |

**Supplementary table 2**. Result from ingenuity pathway analysis

| **No.** | **Pathway Name** | **Total** | **Expected** | **Hits** | **Raw p** | **Impact** |
| --- | --- | --- | --- | --- | --- | --- |
| 1 | alpha-Linolenic acid metabolism | 29 | 0.20482 | 2 | 0.017045 | 0.20335 |
| 2 | D-Glutamine and D-glutamate metabolism | 11 | 0.07769 | 1 | 0.075155 | 0.02674 |
| 3 | Citrate cycle (TCA cycle) | 20 | 0.14125 | 1 | 0.13266 | 0.06327 |
| 4 | Alanine, aspartate and glutamate metabolism | 24 | 0.16951 | 1 | 0.15712 | 0.20703 |
| 5 | Vitamin B6 metabolism | 32 | 0.22601 | 1 | 0.20411 | 0.06044 |
| 6 | Nitrogen metabolism | 39 | 0.27545 | 1 | 0.24319 | 0 |
| 7 | Nicotinate and nicotinamide metabolism | 44 | 0.31076 | 1 | 0.26999 | 0 |
| 8 | Phenylalanine metabolism | 45 | 0.31782 | 1 | 0.27524 | 0 |
| 9 | Lysine degradation | 47 | 0.33195 | 1 | 0.28564 | 0 |
| 10 | Glyoxylate and dicarboxylate metabolism | 50 | 0.35314 | 1 | 0.30097 | 0.00326 |
| 11 | Pyrimidine metabolism | 60 | 0.42376 | 1 | 0.34987 | 0 |
| 12 | Aminoacyl-tRNA biosynthesis | 75 | 0.52971 | 1 | 0.41723 | 0 |
| 13 | Arginine and proline metabolism | 77 | 0.54383 | 1 | 0.4257 | 0 |
| 14 | Purine metabolism | 92 | 0.64977 | 1 | 0.48561 | 0 |
| 15 | Steroid hormone biosynthesis | 99 | 0.69921 | 1 | 0.51151 | 0 |
| 16 | Porphyrin and chlorophyll metabolism | 104 | 0.73452 | 1 | 0.52925 | 0.00954 |

Note: Total is the total number of compounds in the pathway; the Hits is the actually matched number from the user uploaded data; the Raw p is the original p value calculated from the enrichment analysis; the Impact is the pathway impact value calculated from pathway topology analysis.
